# Probing the Role of the Chloroplasts in Heavy Metal Tolerance and Accumulation in *Euglena gracilis*

**DOI:** 10.3390/microorganisms8010115

**Published:** 2020-01-14

**Authors:** Bishal Khatiwada, Mafruha T. Hasan, Angela Sun, Karthik Shantharam Kamath, Mehdi Mirzaei, Anwar Sunna, Helena Nevalainen

**Affiliations:** 1 Department of Molecular Sciences, Macquarie University, Sydney, NSW 2109, Australia; bishal.khatiwada@hdr.mq.edu.au (B.K.); mafruha.hasan@sydney.edu.au (M.T.H.); angela.sun@mq.edu.au (A.S.); karthik.kamath@mq.edu.au (K.S.K.); mehdi.mirzaei@mq.edu.au (M.M.); 2 Biomolecular Discovery and Design Research Centre, Macquarie University, Sydney, NSW 2109, Australia; 3 Australian Proteome Analysis Facility, Macquarie University, Sydney, NSW 2109, Australia

**Keywords:** *Euglena gracilis*, microalga, heavy metal accumulation, mercury accumulator, transmission electron microscopy (TEM), SWATH-MS

## Abstract

The *E. gracilis* Zm-strain lacking chloroplasts, characterized in this study, was compared with the earlier assessed wild type Z-strain to explore the role of chloroplasts in heavy metal accumulation and tolerance. Comparison of the minimum inhibitory concentration (MIC) values indicated that both strains tolerated similar concentrations of mercury (Hg) and lead (Pb), but cadmium (Cd) tolerance of the Z-strain was twice that of the Zm-strain. The ability of the Zm-strain to accumulate Hg was higher compared to the Z-strain, indicating the existence of a Hg transportation and accumulation mechanism not depending on the presence of chloroplasts. Transmission electron microscopy (TEM) showed maximum accumulation of Hg in the cytosol of the Zm-strain and highest accumulation of Cd in the chloroplasts of the Z-strain indicating a difference in the ability of the two strains to deposit heavy metals in the cell. The highly abundant heavy metal transporter MTP2 in the Z-strain may have a role in Cd transportation to the chloroplasts. A multidrug resistance-associated protein highly increased in abundance in the Zm-strain could be a potential Hg transporter to either cytosol or mitochondria. Overall, the chloroplasts appear to have major role in the tolerance and accumulation of Cd in *E. gracilis*.

## 1. Introduction

The use of heavy metals in industrial, medical, household, and agricultural products has led to an increase in the deposition and distribution of these metals in the environment arising serious concerns about their impact on living organisms [1,2]. Different approaches such as application of chemicals have been employed to remove heavy metals from the soil and water but most of these have proven inefficient or expensive. Additional problems are generated by large volumes of contaminated sludge requiring further processing [3]. On the other hand, plants, microorganisms, and microalgae have the ability to bioaccumulate heavy metals sustainably, thus reducing the burden imposed on landfill sites and water resources [4,5].

Phytochelatin-mediated heavy metal detoxification has been previously reported in plants, fungi, and algae [6]. In plants, low molecular weight heavy metals are first complexed with phytochelatins (LMW HM-PC), and transported into vacuoles for the later conversion into high molecular weight heavy metal-phytochelatin complexes (HMW HM-PC). The same mechanism appears to be functional in yeasts and algae [7,8,9,10]. In yeast [11], rice [12], and green algae, mitochondria are also involved in the accumulation of heavy metals [13], where the mitochondrial solute carrier (MSC) family proteins transport solutes and metal ions into the mitochondria [14]. Detoxification of heavy metals in the mitochondria has not reported so far, however, a few studies have indicated that heavy metal accumulation causes metal-induced toxicity and stress in mitochondria [15,16].

Cyanobacteria and photosynthetic green algae have the ability to deposit heavy metals in their chloroplasts [17]. Organisms with chloroplasts have a well-organized cellular machinery for bioaccumulation of metals since some of the cofactors used in the chloroplast electron transfer apparatus, such as iron, copper (Cu) and zinc (Zn) metals are readily available. Following this, toxic heavy metals such as cadmium (Cd), lead (Pb) and mercury (Hg) can mimic essential metals and use their transporters and ion-channels to move inside intracellular organelles [18]. This makes possible the storage of heavy metals inside the chloroplasts; however, if the cells cannot withstand their toxicity, heavy metals are extruded using cellular efflux pumps [19].

In the studies of heavy metal tolerance and accumulation by microalgae such as *Chlamydomonas reinhardtii* [20], *Oedogonium westii* [21] and marine algae [22], bioaccumulation of heavy metals has been mostly observed in the vacuoles [23,24]. *E. gracilis* studied here has the capacity to uptake metals such as Cd, Pb and Hg [24,25] but lacks a proper vacuole found in plants and fungi [26]. Therefore, in the absence of vacuoles, *E. gracilis* might use either the cytoplasm or cellular structures such as chloroplasts and mitochondria to accumulate heavy metals. In a previous study conducted with *E. gracilis* to investigate Cu and Zn accumulation, the results indicated that Cu affected chloroplast organization and metal accumulation was observed inside the contractile vacuole, as indicated by the vacuolar structure. Heavy metals were also distributed into the chloroplasts and cytoplasm [27].

This study was conducted to explore the role of chloroplasts in the accumulation of heavy metals (Cd, Hg and Pb) in *E. gracilis*. The MIC and the maximum ability to accumulate heavy metals were compared between the previously assessed wild-type Z-strain [28] that can develop mature chloroplasts and a variant strain (Zm) that was unable to develop chloroplasts. Sequential window acquisition of all theoretical fragment-ion spectra- mass spectrometry (SWATH-MS) was performed to analyze differences in the proteomes between the two strains. The relative abundance of proteins was compared to identify the proteins involved in heavy metal bioaccumulation. Deposition of the heavy metals inside the cells was established using transmission electron microscopy. 

## 2. Materials and Method

### 2.1. Chemicals

All chemicals and reagents were purchased from Sigma Aldrich (Castle Hill, NSW, Australia) unless stated otherwise.

### 2.2. Algal Strains and Culture Conditions

The *Euglena gracilis* Z-strain (UTEX 753) was obtained from the University of Texas Culture Collection, Austin, TX, USA. A stable non-chloroplast variant *Euglena gracilis* Zm-strain was developed in-house by supplementing the cultivation medium with streptomycin for several generations; this culture has been maintained over two years. The absence of chloroplasts in the Zm-strain was confirmed by the means of a fluorescence microscope, Olympus BX51, using the blue excitation filter (excitation = 480/20, emission = 510LP).

Both strains were grown mixotrophically in a glucose, ammonium chloride and yeast extract (GNY) medium (modified Hutner medium; pH 3.5) [29] at 23 °C with orbital shaking at 150 rpm. The heavy metals mercury (HgCl_2_), lead (Pb (NO_3_)_2_) and cadmium (CdCl_2_) were mixed with the GNY medium in varying concentrations for determination of the minimum inhibitory concentration (MIC) for the Zm-strain. The stock solution of heavy metals was prepared in sterile double distilled water (ddH_2_O) and sterilized using a 0.2-micron filter. All experiments were conducted using biological triplicates.

### 2.3. Minimum Inhibitory Concentration (MIC) Assay

The ability of the *E. gracilis* Zm-strain to withstand heavy metal exposure was determined by the MIC assay as previously described [28]. Briefly, the cultures were subjected to different concentrations of metal: Hg (5–90 ppm), Pb (500–10,000 ppm) and Cd (50–1000 ppm). Cells were counted using a hemocytometer during cultivation. The cell count of cultures treated with heavy metals was compared with that of the untreated cultures, assigned as 100%.

### 2.4. Determination of Heavy Metal Bioaccumulation

The maximum capacity of the *E. gracilis* Zm- strain to bioaccumulate heavy metals was determined using microwave plasma atomic emission spectroscopy (4100 MP-AES, Agilent, USA). Cells were grown in a GNY medium [29] supplemented with either Cd (200 ppm), Pb (500 ppm) and Hg (50 ppm), selected from the MIC study. Samples were collected at different time points (day 2, 4, 6 and 7) of cultivation and heavy metal accumulation was determined as described previously [28].

### 2.5. Transmission Electron Microscopy (TEM)

Both the Z- and Zm- strains grown in a GNY medium supplemented with a particular heavy metal and controls without heavy metal addition were harvested at the log phase by centrifugation (500× *g*; 2 min) and washed twice with ddH_2_O. All chemicals used in TEM were purchased from ProSciTech, Australia. Primary fixation of the cells was performed using 2.5% (*v*/*v*) glutaraldehyde and 2.5% (*v*/*v*) paraformaldehyde for 2 h and secondary fixation was carried out using 2% (*v*/*v*) osmium tetroxide (OsO_4_) for 1.5 h. The cells were dehydrated with graded ethanol solutions (50%, 70%, 90%, 95%, 100% *v*/*v*). After this, the cells were infiltrated with increasing concentrations of London resin (LR) white resin medium grade at 50%, 75% and finally 100% (*v*/*v*). Infiltration was carried out for 2 h for each concentration of LR white resin except the 100% LR white resin, for which the cells were incubated at RT (room temperature) overnight on a rotator. On the following day, the resin was replaced with fresh 100% LR white resin and the cells were incubated at RT for 3 h. The samples were then allowed to polymerize inside an oven at 60 °C for 48 h.

Ultrathin resin sections were prepared on Ultramicrotome Leica EM UC7 and the sections were stained serially with uranyl acetate and lead citrate. The sections were then studied on a Philips CM10 Transmission Electron Microscope. Control cells without metal treatment were compared against heavy metal treated samples to identify the location of heavy metal accumulation.

### 2.6. Extraction of Proteins and Peptides

Aliquots of the *E. gracilis* Zm-strain cultures grown with each heavy metal were collected at the maximum heavy metal accumulation time points as determined by MP-AES (Section 3.2) and proteins and peptides were extracted as described earlier for the Z-strain [28]. All experiments were carried out in biological triplicates.

### 2.7. Mass Spectrometry Analysis

**Figure 1:**
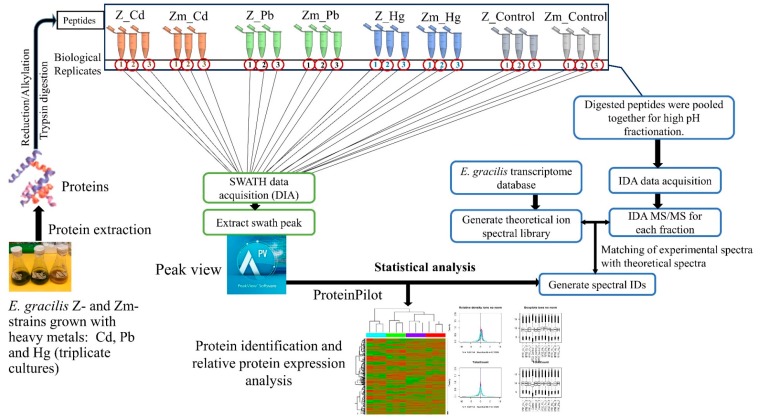
Workflow of SWATH-MS. The empirical ion/spectral library was generated by IDA-MS. SWATH acquisition enabled comparison of the spectra to the spectral library and label-free relative quantification of proteins. The relative abundance of proteins was compared between heavy metal treated and untreated control samples. Statistical analysis was performed as stated in Section 2.9.

A TripleTOF 5600 mass spectrometer was used to analyze the peptides from the proteins extracted from the Zm-strain treated with heavy metals. All experiments were carried out in biological triplicates (Figure 1). The SWATH experiment was carried out in two-steps: information-dependent acquisition-mass spectrometry (IDA-MS) was used to generate the ion library and SWATH-MS was applied for label-free quantification (Figure 1).

### 2.8. Creation of a Peptide Ion Library (IDA-MS)

Extracted peptides were fractionated with modifications as described [28]. The generated ion library was searched against the *Euglena* non-redundant protein database available at the John Innes Centre website (http://jicbio.nbi.ac.uk/euglena/), acquired in September 2016. In the search parameter, carbamidomethylation of cysteine residues was used as a fixed modification. Candidate proteins were identified using the following criteria: *Unused Score* > 2 (signifying 99% confidence level) and global peptide false discovery rate (FDR) < 1%.

### 2.9. SWATH-MS Data Processing and Statistical Analysis

SWATH mass spectrometry and data processing were performed as described previously [28]. A complete SWATH-MS workflow is presented in Figure 1. Peptide extraction, SWATH-MS, and data processing have been carried out with the Z-strain previously [28] and were used here for comparison with the corresponding data obtained from the Zm-strain in this study. Proteins with relative expression fold-changes of ±1.5 and *p*-value < 0.05 were considered statistically significantly altered in expression between respective conditions. Proteomic survey will reveal the proteins involved in heavy metal tolerance and accumulation and the role of chloroplasts and chloroplast-related proteins in heavy metal sequestration and transportation into chloroplasts.

### 2.10. Functional Annotation

The Blast2GO software was used to annotate the proteins [28]. Sequences obtained from the *Euglena* non-redundant proteins database were matched with the UniProtKB/Swiss-Prot database with an *E*-value cut-off of 1e^−10^. Gene Ontology (GO) information of proteins was derived using the UniProt database. 

## 3. Results and Discussion

### 3.1. Fluorescence Microscopy of the E. gracilis Strains

**Figure 2:**
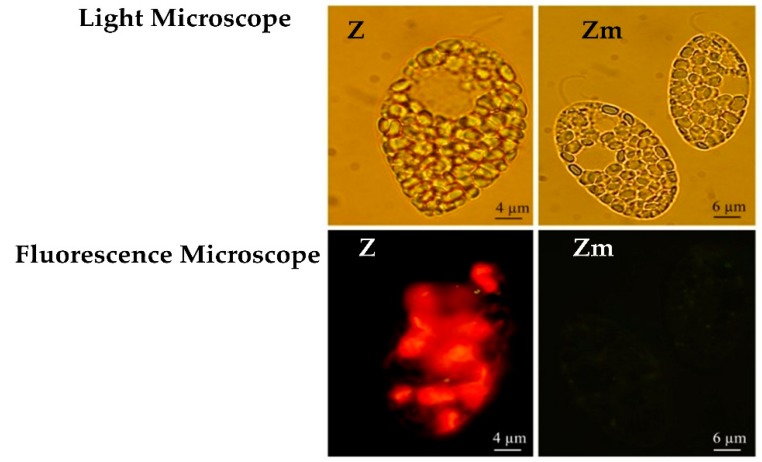
*E. gracilis* strains visualized under the light microscope and fluorescence microscope. Red fluorescence from the chlorophyll in the chloroplasts appears only in the Z-strain.

The loss of chloroplasts in the Zm-strain was confirmed with fluorescence microscopy (Figure 2) and further by TEM (Section 3.3). The wild type Z-strain had a bright red fluorescence brought about by the chlorophyll in the chloroplasts, however, the Zm-strain lacked the red fluorescence (Figure 2). Streptomycin treatment can eliminate chloroplasts from *Euglena* without significantly inhibiting cell division or the viability of the cells [30,31]. 

### 3.2. Minimum Inhibitory Concentration (MIC)

**Figure 3:**
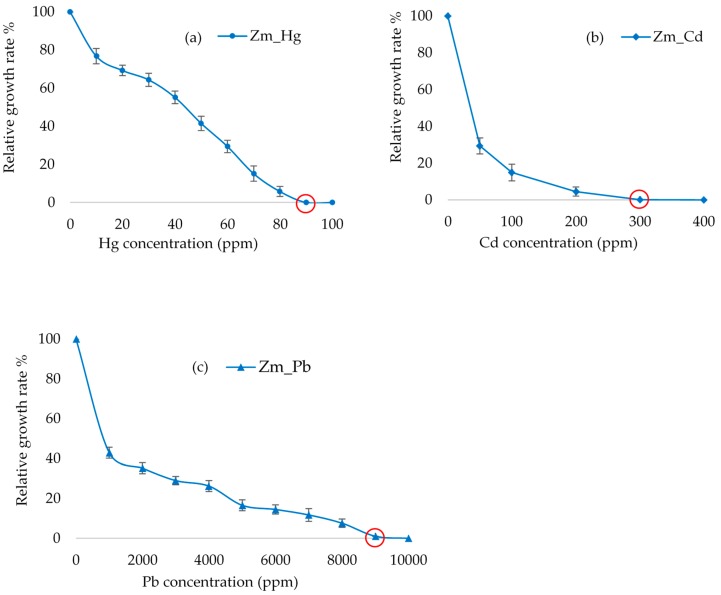
The effect of heavy metals Hg (**a**), Cd (**b**) and Pb (**c**) on the growth of the *E. gracilis* Zm- strain. The graph shows the relative growth of cells against a varying concentration of heavy metals. Error bars represent the standard deviation calculated from biological triplicate samples. Minimum inhibitory concentration (MIC) is indicated by the red circle.

The MIC of the three heavy metals for the Zm-strain varied considerably. The lowest MIC (90 ppm) was observed with Hg, which, however is higher than the Hg MIC of 20 ppm assigned for the freshwater microalgae *Phormidium ambiguum*, *Pseudochlorococcum typicum* and *Scenedesmus quadricauda* var *quadrispina* [32]. Hg tolerance of 71 ppm has been recorded for brown algae [33]. Although the *E. gracilis* Zm-strain lacking chloroplasts had a slightly higher Hg tolerance when compared to that of the Z-strain (80 ppm) [28], the relatively small difference and non-existing information available for other algae do not allow drawing sound conclusions for the possible role of the chloroplasts in Hg tolerance in algae. The MICs of the Z-strain [28] and Zm-strain (Figure 3) were brought about by the difference in the cellular ability of the strains to withstand the toxicity of each heavy metal since the growth rates between the untreated Z- and Zm- strains were not significantly different (*p* > 0.05) (Supplementary Figure S1).

The MIC for Pb in the Zm-strain was 9000 ppm, which is about the same as that of the Z-strain from our previous study [28]. Thus, similarly to Hg tolerance, chloroplasts may not be involved in the tolerance to Pb. Differently to the about similar Hg and Pb MIC values established for the two strains, the MIC for Cd with the Zm-strain lacking chloroplasts was 300 ppm, which is about half of the 600 ppm determined earlier for the Z-strain containing chloroplasts [28]. A lower tolerance to Cd in the Zm-strain lacking chloroplasts may indicate a role for these organelles in Cd tolerance in *Euglena*.

### 3.3. Evaluation of the Heavy Metal Accumulation

MP-AES analysis of the Zm-strain showed that it could accumulate Hg in higher amounts, 6.54 mg/g dry weight (DW) at day 2 compared to Cd and Pb. The maximum Cd accumulation was recorded on day 6 (4.14 mg/g DW) while only 1.93 mg/g DW accumulation of Pb was observed on day 4.

**Figure 4:**
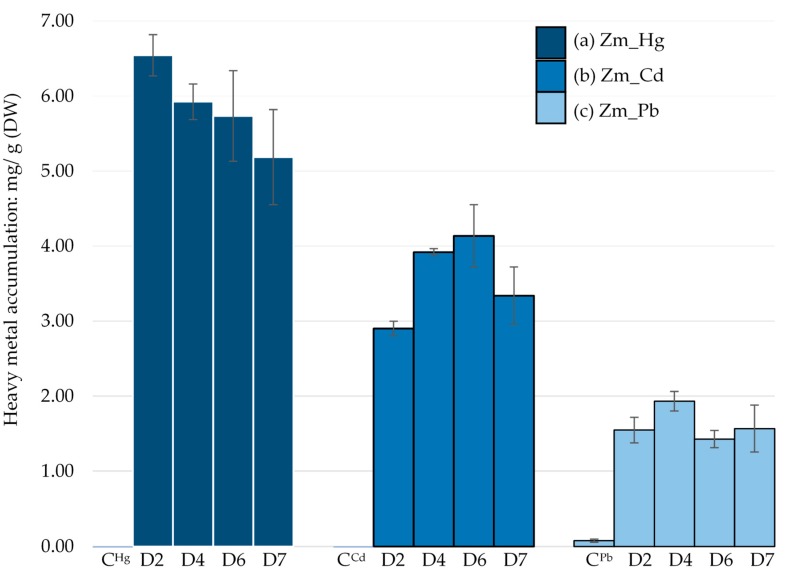
*E. gracilis* Zm-strain treated with different heavy metals: (**a**) Hg, (**b**) Cd and (**c**) Pb. C^Hg^, C^Cd^ and C^Pb^ were untreated controls. Heavy metal accumulation per gram of dry weight of each sample was measured between day 2 and 7 by MP-AES. Error bars represent the standard deviation calculated from the biological triplicate samples.

Although Hg is more toxic than Cd and Pb, the order of heavy metal bioaccumulation by the Zm-strain was Hg > Cd > Pb (Figure 4). This suggests that the Zm-strain has an ability to sequester Hg into a non-toxic form even in the absence of chloroplasts. In our previous study, the average Hg accumulation from day 2 to day 7 by the wild type Z-strain was 3.77 mg/g (DW) [28], which is comparatively lower than that of the Zm-strain (5.84 mg/g, DW) under similar conditions. 

The average Cd accumulation by the Zm-strain was 3.5 mg/g (DW), which is lower than in the Z-strain (5.23 mg/g of DW) [28]. This suggests that chloroplasts may contribute to Cd bioaccumulation. Cd accumulation in the chloroplasts has been reported for *Hordeum vulgare*, *Zea mays* [34] and a few other plants [35]. Although some studies are currently underway to understand the role of chloroplasts in Cd accumulation, most studies have been dedicated to reveal the effects of Cd toxicity on inhibition of photosynthesis [36,37]. 

**Figure 5:**
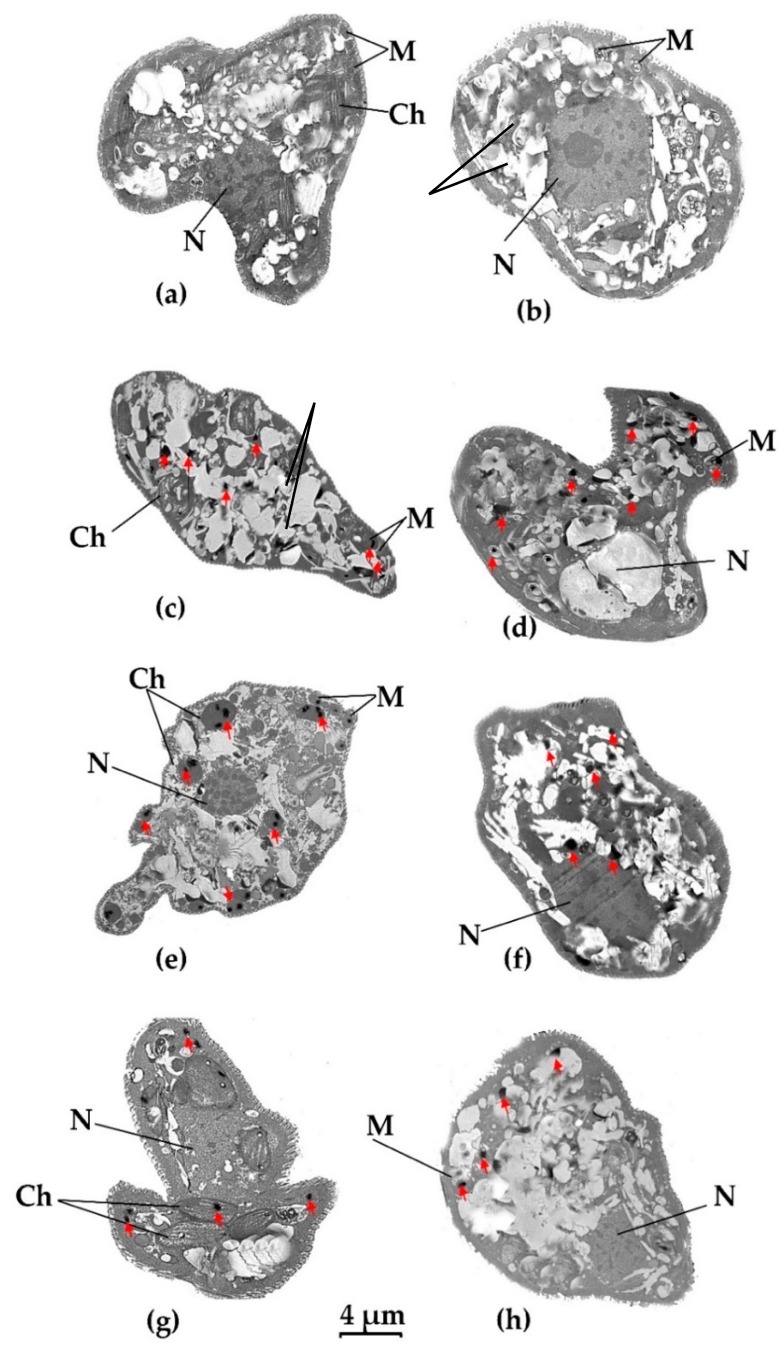
Visualization of heavy metal accumulation in *E. gracilis* strains using transmission electron microscope (TEM). (**a**) Z-strain control- untreated cell; (**b**) Zm-strain control- untreated cell; (**c**): Z-strain exposed to Hg; (**d**): Zm-strain exposed to Hg; (**e**): Z-strain exposed to Cd; (**f**): Zm-strain exposed to Cd; (**g**): Z-strain exposed to Pb; (**h**): Zm-strain exposed to Pb. P: Paramylon; N; nucleus, M; mitochondria, Ch; chloroplast. Black dots represent heavy metal inclusion and are marked by a red arrow. The 4 µm bar corresponds to all images.

The least amount of heavy metal accumulation by the Zm-strain was for Pb, which remained almost constant between day 2 and 7. MIC results indicated that Pb was also the least toxic of the three metals tested (Figure 4). The average Pb bioaccumulation was 1.62 mg/g (DW), which is similar to the 1.82 mg/g (DW) by the Z-strain [28]. As the difference between the accumulation of Pb by chloroplast and non-chloroplast-containing strains was not significant, chloroplasts may not greatly contribute to Pb bioaccumulation. Despite its lower toxicity, Pb has been reported to cause inhibition of the photosystem in plants, leading to swollen and deformed chloroplasts [38,39]. In the alga *Chara vulgaris*, Pb impaired the membrane system of chloroplasts and destroyed the thylakoid structure [40]. However, Pb does not appear to impair the chloroplasts of *E. gracilis* (Figure 5g).

### 3.4. Visualization of Heavy Metal Bioaccumulation

TEM was used to locate heavy metal bioaccumulation in different cellular organelles in the two *E. gracilis* strains. Treated cells were compared against control cells without heavy metal treatment. Ultrathin sections revealed the internal structure of the cell more clearly in the control cells, while those treated with heavy metals seemed to have lost some cellular integrity. TEM images in Figure 5 show recognizable cellular structures such as nuclei (N), mitochondria (M) and chloroplasts (Ch), and the “ridges and grooves” pattern of the pellicle (cell wall). As expected, there were no chloroplasts in the Zm-strain. The accumulated heavy metals appeared as small black dots while aggregated paramylon granules appeared as white round and oval structures. *Euglena* does not have a designated reservoir organelle similar to vacuoles in plants [41].

The chloroplasts of *E. gracilis* are generally spindle-shaped [42]. In the Z-strain treated with Pb, the long spindle-shaped chloroplasts were clearly visible (Figure 5g) while most of the chloroplast structures were damaged in the Hg-treated Z-strain cells, probably because of its toxicity (Figure 5c). The shape of the chloroplasts in the Cd-treated cells of the Z-strain were affected (not spindle shape), probably due to Cd bioaccumulation and toxicity (Figure 5e). The Zm-strain lacked visible chloroplasts.

In the Z-strain, accumulation of Hg took place in the cytosol and mitochondria (Figure 5c); a similar deposition pattern was also observed for the Zm-strain (Figure 5d). However, it appears that the number of black dots representing Hg accumulation was higher in the Zm-strain than in the Z-strain. This result correlates with the fact that the Zm-strain was able to accumulate more Hg compared to the Z-strain (Figure 4) [28]. This also suggests that chloroplasts do not play a major role in Hg accumulation or the chloroplasts in the Z-strain were damaged by Hg resulting in a decrease in their ability to take up the metal. Damage to the chloroplast by mercury has been reported previously in plants and the green alga *Chara vulgaris* [40,43].

Cd accumulation mostly occurred in the chloroplasts in the Z-strain, although Cd accumulation was also found in the cytosol and mitochondria (Figure 5e). In the Zm-strain, Cd deposition was seen mostly in the cytosol (Figure 5f). The Z-strain accumulated more Cd than the Zm-strain which suggests that chloroplasts may have a role in Cd accumulation in the Z-strain. The maximum bioaccumulation was observed during Cd treatment of the Z-strain, suggesting an established mechanism to sequester large amounts of Cd in the chloroplasts, rendering this strain a Cd hyperaccumulator [28].

Pb accumulation was the least among the heavy metals studied here and is consistent with Pb accumulation shown in Figure 4. Pb accumulation was distributed fairly evenly in the chloroplasts and cytosol of the Z-strain (Figure 5g). The Zm-strain was found to accumulate Pb in the cytosol and mitochondria. Pb accumulation may have been assisted by chloroplasts in the Z-strain, as some black dots were observed in these organelles although both the Z- and Zm-strains had an almost similar ability to accumulate Pb. Chloroplasts seem to be intact and not deformed by Pb compared to the negative effect of Hg, which highlights the different effects of these heavy metals on cellular organelles of *E. gracilis*.

The preliminary data obtained from MIC studies, metal accumulation and heavy metal localization revealed differences in the ability and mode of the two strains to handle Hg, Pb, and Cd. 

### 3.5. Proteome Profiling of Zm-Strain in Response to Heavy Metals Using SWATH-MS

Differentially abundant proteins during heavy metal treatment provide a basis to understand the molecular mechanism of heavy metal tolerance and bioaccumulation. The differentially abundant proteins in the Zm-strain exposed to heavy metals were compared to the control without heavy metal exposure. The differentially abundant proteins of the previously assessed Z-strain [28] and the Zm- strain identified in this study were also compared. Through a SWATH-MS analysis, a total of 5325 proteins with FDR < 1% were identified across the strains and heavy metal treatments, and 4493 proteins were quantified.

Proteome profile of the *E. gracilis* Zm-strain was significantly changed after exposure to the heavy metals Cd, Pb and Hg when compared to the untreated control of the same strain (Table 1). 

**Figure 6:**
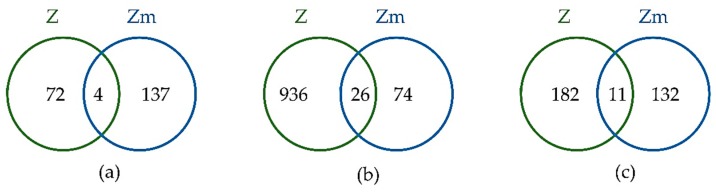
Venn diagram comparing the differentially abundant proteins between *E. gracilis* Z-and Zm-strains when treated with the heavy metal Hg (**a**), Cd (**b**), and Pb (**c**). The intersection of the sets represents the differentially abundant proteins common to both strains for the respective heavy metal treatment.

The number of differentially abundant proteins in the Hg-treated Zm-strain was 141, which is higher than the Z-strain (76 proteins) [1]. Despite the high level of toxicity of Hg compared to the other two heavy metals, the Zm-strain was able to accumulate large quantities of Hg (5.84 mg/g, DW). A larger number of differentially abundant proteins resulting from Hg exposure indicated a substantial involvement of proteins to either defend the Hg toxicity or regulate the cellular machinery to accumulate it for further detoxification. During the Cd treatment, 960 proteins were increased in abundance (FC > 1.5) in the Z-strain [28], compared to just 100 proteins in the Zm-strain. This vast difference in the number of differentially abundant proteins indicates that the chloroplasts in the Z-strain may play a major role in Cd accumulation and detoxification. Cd accumulation by the Z-strain was the highest among the heavy metals studied here and was also higher than that of the Zm-strain. Pb exposure returned a similar number of differentially abundant proteins for both strains, which is reflective of the similar MIC and accumulation patterns (Figure 6). 

The differentially abundant proteins between the two strains were compared to identify whether common or unique proteins were involved in heavy metal tolerance and accumulation (Figure 6).

Among the 213 differentially abundant proteins during Hg treatment, only 4 proteins (1.87%) were common to both strains, while for the Cd and Pb treatments 26 proteins (2.51%) and 11 proteins (3.38 %) out of a total of 1034 and 325 differentially abundant proteins were common, respectively. This small percentage of common differentially abundant proteins between the strains suggests that strains with and without chloroplasts have different mechanisms for heavy metal tolerance and accumulation. The complete list of differentially abundant proteins common to both strains is shown in Supplementary Table S1.

#### 3.5.1. Gene Ontology (GO) Annotation

**Figure 7:**
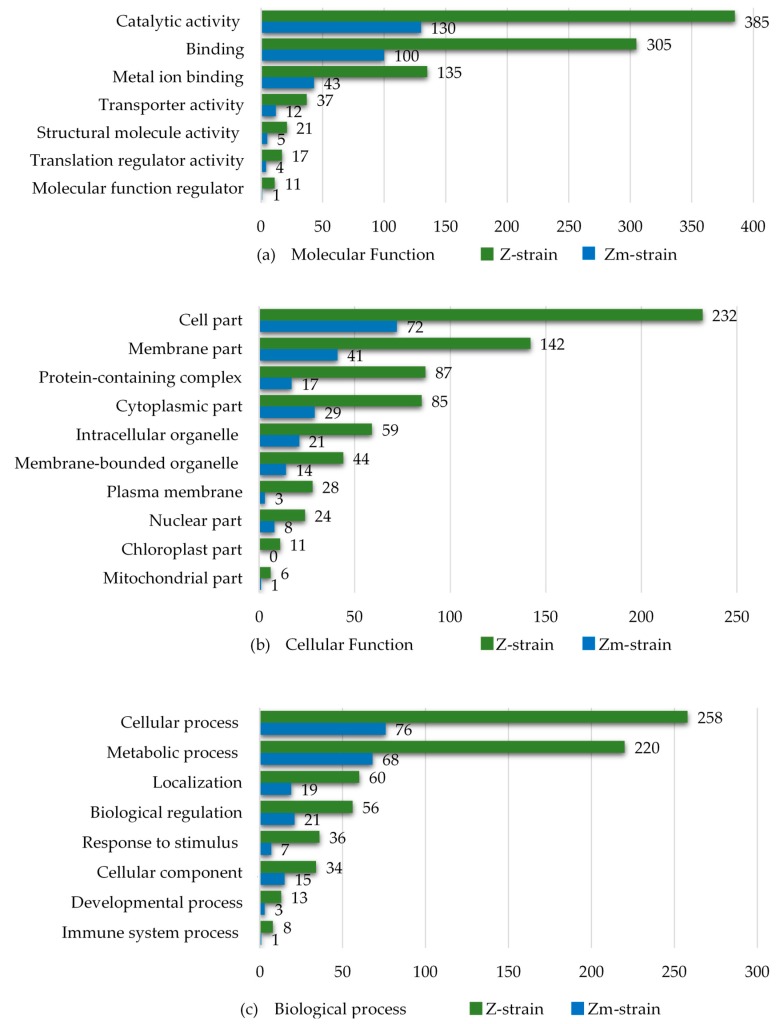
Gene Ontology (GO) annotation of the differentially abundant proteins of the *E. gracilis* Z- and Zm- strains treated with the heavy metal Cd, Hg, and Pb (one metal at a time). The results were collated into three sets: (**a**) Molecular function, (**b**) Cellular component and (**c**) Biological process.

The differentially abundant proteins from the Hg, Cd and Pb treatments were categorized by their GO annotations using the UniProt database and plotted collectively (Figure 7). 

**Figure 8:**
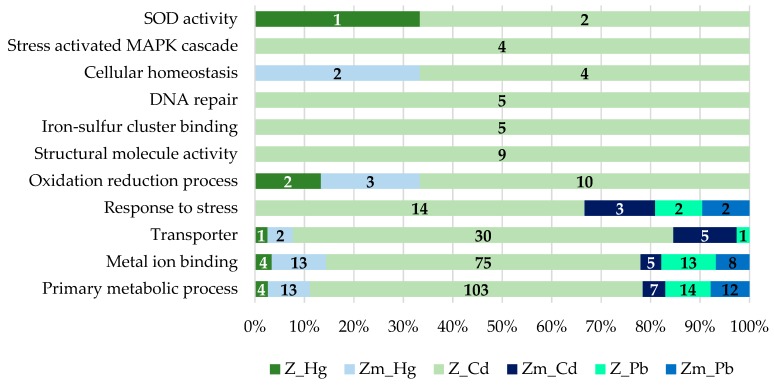
GO annotation of the proteins increased in abundance (fold change ±1.5, *p* < 0.05) in *E. gracilis* Z- and Zm- strains treated with the heavy metals Cd, Hg, and Pb. The proteins were categorized into different sets and denoted by bars specified with numbers (normalized to the total number).

The number of differentially abundant proteins in the categories of “metal ion binding” and “transporter activity” were about three times higher in the Z-strain compared to the Zm-strain. The results also revealed that in all subcategories of the set “Cellular components”, the Z-strain showed an extensive response towards heavy metal exposure. The Z-strain appeared metabolically very active, most probably because of the presence of chloroplasts and performing photosynthesis. The number of differentially abundant proteins in the Z-strain were higher in both the cytosol and intracellular organelles. As expected, no chloroplast-related proteins were found in the Zm-strain. In the set “Biological process”, the Z-strain was more responsive in the subcategories of “cellular” and “metabolic process” during exposure to heavy metals. This suggests that the Z-strain was more reactive towards external stimuli, such as heavy metal toxicity, producing stress responses during exposure. To further understand the ability of the strains to cope with metal toxicity, the proteins increased in abundance in each type of metal treatment were compared (Figure 8).

The proteins increased in abundance, which may have a role in heavy metal bioaccumulation were categorized into different sets. The proteins increased in abundance in the Zm-strain are listed in Supplementary Table S2 and the data of proteins increased in abundance in the Z-strain was sourced from our previous study [28]. The analysis showed that during the Hg treatment, the number of proteins involved in the primary metabolic processes was lower in the Z-strain compared to the Zm-strain. Heavy metal ions can disrupt the primary metabolic function in several ways such as by damaging organelles or by replacing the essential metals in metalloproteins, particularly those that carry out enzymatic reactions. A drastic change in the proteome profile of the Z-strain treated with Cd compared to the Zm-strain was observed (Figure 8). This provides further evidence that the chloroplasts in *E. gracilis* assist to develop high tolerance against Cd and may have a proper mechanism to accumulate Cd.

The induction of metal ion binding proteins occurs in conjunction with various metals and the active inducers are Cd, Zn, Pb, silver (Ag) and antimony (Sb) [44]. In our study, the metal-binding proteins were highly abundant in the Z-strain during Cd exposure. Hg and Pb exposure resulted in very few changes in the abundance of metal-binding proteins in both strains. Metal-binding proteins have specific binding abilities to different metals depending on the organism and strain. For instance, the Zm-strain lacks chloroplasts and its ability to bind and accumulate Cd was lower compared to the Z-strain. In addition, the Zm-strain had more specificity for Hg than Cd. Metal-binding proteins also play an important role in the intracellular trafficking of metal ions. Metal-binding proteins such as glutathione synthetase, second enzyme in the glutathione (GSH) biosynthesis pathway, was increased by 14-fold in the Cd-treated Z-strain but not in the Hg and Pb-treated cells (Supplementary Table S3). Glutathione is crucial for heavy metal chelation and subsequent bioaccumulation in intracellular organelles. The number of several transition metal ion binding proteins was significantly enhanced during Cd treatment of the Z-strain. The increase in the GSH during Cd treatment in the chloroplast containing strain indicates the cellular ability to accumulate them in the chloroplast as shown by TEM studies (Figure 5e) and sequester them to non-toxic form.

Damage on DNA caused by heavy metals includes breaks in the double-stranded molecules as well as leads to inhibition of some of the critical proteins of the DNA repair pathway [45]. The level of DNA damage depends on the toxicity of the respective heavy metal and its concentration. Most importantly, the cellular ability to initiate the response towards DNA repair and the choice of construction or repair pathway determine the cellular ability to accumulate heavy metals [45]. In our study, five proteins responsible for initiating DNA repair were highly abundant in only the chloroplast containing Z-strain during Cd treatment compared to none in the non-chloroplast Zm-strain (Figure 8). This may indicate the ability of the chloroplast containing strain to bioaccumulate and sequester Cd to protect against DNA damage. 

#### 3.5.2. Transporters

Transporters have a significant role in metal homeostasis. There were approximately five times more transporter proteins of which relative abundance was increased during heavy metal treatments in the Z-strain than in the Zm-strain (Table 2) and most of the changes in their abundance in the Z-strain were caused by Cd treatment. Various heavy metal transporters have been characterized in plants [46,47] and yeast [48,49] and green alga *Chlamydomonas* [14,50]. The highly abundant transporters in the chloroplast and non-chloroplast containing strains were different except the ABC transporter. Transporters in the Zm-strain may have a role in the intracellular trafficking of heavy metals and in the absence of chloroplasts, the most likely destination of heavy metal deposition would be either the mitochondria or cytosol. Two transporters were higher in abundance during Hg treatment of the Zm-strain (Table 2), including the copper-transporting ATPase RAN1. Copper-ATPases are crucial for maintaining metal homeostasis [51]. A transmembrane transporter multidrug resistance-associated protein member 2 (MRP2) was increased by 11.82-fold in the Zm-strain. MRP2 is involved in the transport of heavy metal conjugates into cellular organelles from the cytosol via ATP-dependent export pumps [52]. This transporter may be involved in Hg transport from the cytosol to mitochondria in the Zm-strain. Moreover, the ABC transporter G family member 36, which has been characterized as a probable efflux pump of heavy metal ions [53], was also higher in abundance in the Cd treated Zm-strain. Overexpression of this transporter has made plants more resistant to heavy metals [53]. The Zm-strain may thus have also used this transporter to efflux the Cd and Pb out of the cells. 

In the Z-strain, the abundance of transporter proteins was most increased after Cd-treatment compared to other heavy metals whereas in the Zm-strain Hg brought the highest increase in the abundance of transporter proteins (Figure 8; Table 2). The increase in abundance of different types and numbers of transporter proteins in the two strains highlights the difference in the molecular mechanism of chloroplast and non-chloroplast strains to tolerate or accumulate various heavy metals. Transporters that play a role in intracellular metal transportation were found to be highly abundant in the Z-strain. The cation diffusion facilitator (CDF) family of transporters are well known to chelate the heavy metals and sequester them to cellular organelles like the vacuole [54]. In chloroplast-containing plants, CDFs are known as Metal Tolerance Proteins (MTPs) because of their role in the sequestration of excessive Zn in the vacuoles [55]. MTP2, which was previously characterized as a transporter that has a role in Zn hyperaccumulation in *Arabidopsis halleri* and *Noccaea caerulescens* [55], was found to be highly abundant, by 3.61-fold increase, in the Z-strain (Table 2). Since *E. gracilis* lacks a plant-like proper vacuolar structure, the MTP2 transporter in the Z-strain may be involved in Cd sequestration into the chloroplasts, although the functional characterization of this transporter has not been performed yet. The increase in abundance of a P-type ATPase was 3.6-fold in the Z-strain. P-type ATPases are also known as heavy metal ATPases (HMAs) that are associated with the transport of heavy metals in plants [56] and yeast [57]. P-type ATPase are localized to the vacuolar membrane and helps in detoxification of Zn and Cd through vacuolar sequestration in yeast [58]. In *Arabidopsis thaliana* P-type ATPase are localized to the chloroplasts and assist in Zn detoxification [59]. The increase in abundance of the ABC transporter G family member 36 was 4.41-fold (Table 2) in the Z-strain treated with Hg; the Z-strain may have used this transporter to efflux the toxic Hg out of the cell similar to the probable efflux of Cd used by the Zm-strain above. Overall, the transporter study indicated that chloroplasts have a role in bioaccumulation of Cd and efflux of Hg in the Z-strain. Although the Zm-strain lacks chloroplasts it showed an excellent capacity to bioaccumulate Hg suggesting a role for the transporters in Hg accumulation and efflux of Cd.

#### 3.5.3. Chloroplast Proteins

A few chloroplast proteins were high in abundance in the Z-strain treated with Cd, while Hg and Pb did not bring about any significant changes in the chloroplast proteins. The Light-harvesting chlorophyll a/b binding protein (LHCB) was increased by 15.35-fold in the Z-strain (Table 3). LHCB expression operates a special mechanism in plants to modulate chloroplast stomatal movement against cellular stress caused by external stimuli such as exposure to heavy metals and drought; overall, LHCB functions to maintain plant fitness [60,61]. Cd is a strong inhibitor of photosynthesis and interrupts the electron transport chain in photosystem I (PSI) and photosystem II (PSII) [62]. The increase in expression of LHCB may help to better deal with the Cd toxicity and cope with the cellular stress brought by it. The level of Cd toxicity and cellular ability to resist it varies among different organisms. In our study, major changes in the abundance of chloroplast related proteins were seen in the Cd-treated Z-strain. As none of these proteins showed similar levels of changes during the other metal exposures, chloroplast related proteins may be specifically helping the Z-strain to withstand the toxicity of Cd.

## 4. Conclusions

*E. gracilis* chloroplasts were found to have a role in assisting the accumulation of the heavy metal Cd, whereas the other two metals tested, Hg and Pb, were mostly deposited in the cytosol of both strains studied. Some deposition of Hg was also seen in the mitochondria. This, together with the proteomic data, indicates that the strains with (Z) and without chloroplasts (Zm) have different abilities and mechanisms to respond to the heavy metal exposure, especially concerning Cd and Hg. Major changes in the abundance of chloroplast related proteins were seen in the Cd-treated Z-strain including light-harvesting chlorophyll a /b binding protein of PSII, indicating that the chloroplast related proteins may be specifically helping the Z-strain to withstand the toxicity of Cd. Exposure to Hg caused disturbance in the cell integrity, especially in the chloroplasts; however, Cd and Pb exposure did not bring about significant changes in cellular structure. While we focused on protein transporters and chloroplast-associated proteins, further analysis of all identified proteins will add to the current knowledge on the molecular mechanism operating in the tolerance and accumulation of heavy metals in *E. gracilis.* Considering the current data and the fact that *E. gracilis* has an innate tolerance to relatively high concentrations of heavy metals as demonstrated by the MICs obtained, the organism has the potential for the management of particular heavy metal contaminations of the environment.

## Figures and Tables

**Table 1:** Summary of proteins identified from *E. gracilis* Zm-strain. Triplicates were used for all experiments.

Heavy Metal Exposure	Differentially Abundant Proteins *	Increased in Abundance (>1.5 FC *)	Decreased in Abundance (<1.5 FC *)
**Hg**	141	63	78
**Cd**	100	64	36
**Pb**	143	51	92

* (FC) Fold change ±1.5, *t*-test *p*-value < 0.05.

**Table 2:** The highly abundant transporters (FC > 2) during the Cd, Hg, and Pb treatments in *E. gracilis* Z- and Zm-strains.

UniProt ID	Transporter	Heavy Metal	Fold Change
**Zm-strain**			
F1DB26	Multidrug resistance-associated protein_2	Hg	11.82
A0A2R5GHD4	Copper-transporting ATPase RAN1	Hg	7.59
Q9XIE2	ABC transporter G family member 36	Cd	4.52
D0P1Q1	Transmembrane transporter	Cd	2.02
**Z-strain**			
D7LF89	Heavy metal transporter MTP2	Cd	5.61
Q9XIE2	ABC transporter G family member 36	Hg	4.41
Q8H384	Cadmium/zinc-transporting ATPase	Cd/Pb	3.64/2.45
A0A1F3CY04	Copper-translocating P-type ATPase	Cd	3.60
D7FUC2	Ion transmembrane transporter activity	Cd	3.46
A0A1A0FLX3	Potassium transporter TrkA	Cd	3.36
W2ZAX1	Cation-transporting ATPase activity	Cd	3.25
K8EBM6	Proton-transporting ATP synthase activity	Cd	2.95
A0A1E4SU43	Mitochondrial carrier family	Cd	2.87
Q6BZ66	Transmembrane transporter	Cd	2.80
A0A140AY28	Sodium/potassium-transporting ATPase	Cd	2.73
K4ENZ7	Calcium-transporting ATPase	Cd	2.62
A0A261XUE4	Cation-transporting ATPase activity	Cd	2.50
Q9XIE2	ABC transporter G family member 36	Hg	2.41
V4AGW4	Extracellular ligand-gated ion channel	Cd	2.34
E6Y2N7	ATP: ADP antiporter activity	Cd	2.28
E1B2R7	ATP-binding cassette sub-family	Cd	2.26
Q9XIE2	ABC transporter G family member 36	Cd	2.20
Q4DK78	V-type proton ATPase subunit a	Cd	2.00

**Table 3:** The chloroplast related proteins increased in abundance in the Z-strain treated with Cd.

Entry	Protein Name	Fold Change
Q39725	Light-harvesting chlorophyll a /b binding protein of PSII	15.35
A4QPI2	Chloroplast light-harvesting complex II protein	8.17
A8HPC6	Chloroplast light-harvesting complex I protein	6.63
P12356	Photosystem I reaction center subunit III	5.99
A0A061RZ43	Proton gradient regulation 5	4.47
Q8GZR2	Cytochrome f, chloroplastic	4.28
A8JH60	Predicted protein chloroplastic	2.92
Q06SJ8	Photosystem I assembly protein Ycf3	2.84
A8IRG9	Predicted protein chloroplastic	1.81

## Supplementary Materials

The following are available online at https://www.mdpi.com/2076-2607/8/1/115/s1, Figure S1: *E. gracilis* Z- and Zm- strains cultured in the GNY-medium only. Cells were collected each day until day 7. Error bars represent the standard deviation calculated from biological triplicates. The difference in the growth rate observed between the strains were not significantly different (*p* > 0.05). Table S1: Common differentially abundant proteins in the *E. gracilis* Z- and Zm- strains treated with heavy metals Cd, Hg and Pb. Table S2: The high abundance proteins in *E. gracilis* Zm-strain during exposure to the heavy metals Cd, Pb and Hg. Table S3: The high abundance metal binding proteins (FC >1.5) in the *E. gracilis* Z- and Zm- strains treated with heavy metals.

## References

[1] Järup L. (2003). Hazards of heavy metal contamination. Br. Med..

[2] Johri N., Jacquillet G., Unwin R. (2010). Heavy metal poisoning: The effects of cadmium on the kidney. Biometals.

[3] Juang R.-S., Lin L.-C. (2000). Treatment of complexed copper (II) solutions with electrochemical membrane processes. Water Res..

[4] Pulford I., Riddell-Black D., Stewart C. (2002). Heavy metal uptake by willow clones from sewage sludge-treated soil: The potential for phytoremediation. Int. J. Phytoremediation.

[5] Yoon J.M., Oliver D.J., Shanks J.V. (2007). Phytotoxicity and phytoremediation of 2, 6-dinitrotoluene using a model plant, *Arabidopsis thaliana*. Chemosphere.

[6] Schat H., Llugany M., Vooijs R., Hartley-Whitaker J., Bleeker P.M. (2002). The role of phytochelatins in constitutive and adaptive heavy metal tolerances in hyperaccumulator and non-hyperaccumulator metallophytes. J. Exp. Bot..

[7] Hasan M.K., Cheng Y., Kanwar M.K., Chu X.-Y., Ahammed G.J., Qi Z.-Y. (2017). Responses of Plant Proteins to Heavy Metal Stress—A Review. Front. Plant Sci..

[8] Luo Z.-B., Wu C., Zhang C., Li H., Lipka U., Polle A. (2014). The role of ectomycorrhizas in heavy metal stress tolerance of host plants. Environ. Exp. Bot..

[9] Mishra S., Dubey R. (2006). Heavy metal uptake and detoxification mechanisms in plants. Int. J. Agric..

[10] Kneer R., Kutchan T.M., Hochberger A., Zenk M.H. (1992). *Saccharomyces cerevisiae* and *Neurospora crassa* contain heavy metal sequestering phytochelatin. Arch. Microbiol..

[11] Mühlenhoff U., Stadler J.A., Richhardt N., Seubert A., Eickhorst T., Schweyen R.J., Lill R., Wiesenberger G. (2003). A specific role of the yeast mitochondrial carriers MRS3/4p in mitochondrial iron acquisition under iron-limiting conditions. J. Biol. Chem..

[12] Bashir K., Ishimaru Y., Shimo H., Nagasaka S., Fujimoto M., Takanashi H., Tsutsumi N., An G., Nakanishi H., Nishizawa N.K. (2011). The rice mitochondrial iron transporter is essential for plant growth. Nat. Commun..

[13] Nouet C., Motte P., Hanikenne M. (2011). Chloroplastic and mitochondrial metal homeostasis. Trends Plant Sci..

[14] Blaby-Haas C.E., Merchant S.S. (2012). The ins and outs of algal metal transport. BBA-Mol. Cell Res..

[15] Belyaeva E.A., Dymkowska D., Więckowski M.R., Wojtczak L. (2008). Mitochondria as an important target in heavy metal toxicity in rat hepatoma AS-30D cells. Toxicol. Appl. Pharmacol..

[16] Ranjbar A., Ghasemi H., Rostampour F. (2014). The role of oxidative stress in metals toxicity; mitochondrial dysfunction as a key player. Galen Med. J..

[17] Shcolnick S., Keren N. (2006). Metal homeostasis in cyanobacteria and chloroplasts. Balancing benefits and risks to the photosynthetic apparatus. Plant Physiol..

[18] Marchetti C. (2013). Role of calcium channels in heavy metal toxicity. ISRN Toxicol..

[19] Nies A.T., Keppler D. (2007). The apical conjugate efflux pump ABCC2 (MRP2). Pflugers Arch.

[20] Hanikenne M. (2003). Chlamydomonas reinhardtii as a eukaryotic photosynthetic model for studies of heavy metal homeostasis and tolerance. New Phytol..

[21] Shamshad I., Khan S., Waqas M., Asma M., Nawab J., Gul N., Raiz A., Li G. (2016). Heavy metal uptake capacity of fresh water algae (*Oedogonium westti*) from aqueous solution: A mesocosm research. Int. J. Phytoremediation.

[22] Yu Q., Matheickal J.T., Yin P., Kaewsarn P. (1999). Heavy metal uptake capacities of common marine macro algal biomass. Water Res..

[23] Pawlik-Skowrońska B. (2003). Resistance, accumulation and allocation of zinc in two ecotypes of the green alga Stigeoclonium tenue Kütz. coming from habitats of different heavy metal concentrations. Aquat Bot..

[24] Devars S., Hernandez R., Moreno-Sánchez R. (1998). Enhanced heavy metal tolerance in two strains of photosynthetic *Euglena gracilis* by preexposure to mercury or cadmium. Arch. Environ. Contam. Toxicol..

[25] Moreno-Sánchez R., Rodríguez-Enríquez S., Jasso-Chávez R., Saavedra E., García-García J.D., Schwartzbach S.D., Shigeoka S. (2017). Biochemistry and Physiology of Heavy Metal Resistance and Accumulation in *Euglena*. Euglena: Biochemistry, Cell and Molecular Biology.

[26] Mendoza-Cózatl D.G., Moreno-Sánchez R. (2005). Cd^2+^ transport and storage in the chloroplast of *Euglena gracilis*. Biochim. Biophys. Acta.

[27] Einicker-Lamas M., Mezian G.A., Fernandes T.B., Silva F.L., Guerra F., Miranda K., Attias M., Oliveira M.M. (2002). *Euglena gracilis* as a model for the study of Cu^2+^ and Zn^2+^ toxicity and accumulation in eukaryotic cells. Environ. Pollut..

[28] Khatiwada B., Hasan M.T., Sun A., Kamath K.S., Mirzaei M., Sunna A., Nevalainen H. (2020). Proteomic response of *Euglena gracilis* to heavy metal exposure–Identification of key proteins involved in heavy metal tolerance and accumulation. Algal Res..

[29] Rodríguez-Zavala J., Ortiz-Cruz M., Mendoza-Hernández G., Moreno-Sánchez R. (2010). Increased synthesis of α-tocopherol, paramylon and tyrosine by *Euglena gracilis* under conditions of high biomass production. J. Appl. Microbiol..

[30] Ebringer L., Mego J., Jurášek A., Kada R. (1969). The action of streptomycins on the chloroplast system of *Euglena gracilis*. Microbiology.

[31] Ben-Shaul Y., Ophir I. (1970). Effects of streptomycin on plastids in dividing *Euglena*. Planta.

[32] Shanab S., Essa A., Shalaby E. (2012). Bioremoval capacity of three heavy metals by some microalgae species (Egyptian Isolates). Plant. Signal. Behav..

[33] Esmaeili A., Saremnia B., Kalantari M. (2015). Removal of mercury (II) from aqueous solutions by biosorption on the biomass of *Sargassum glaucescens* and *Gracilaria corticata*. Arab. J. Chem..

[34] Lysenko E.A., Klaus A.A., Pshybytko N.L., Kusnetsov V.V. (2015). Cadmium accumulation in chloroplasts and its impact on chloroplastic processes in barley and maize. Photosynth. Res..

[35] Hakmaoui A., Ater M., Boka K., Baron M. (2007). Copper and cadmium tolerance, uptake and effect on chloroplast ultrastructure. Studies on *Salix purpurea* and *Phragmites australis*. Z. Naturforsch. C.

[36] Lee H.-Y., Back K. (2017). Cadmium disrupts subcellular organelles, including chloroplasts, resulting in melatonin induction in plants. Molecules.

[37] Weigel H.J. (1985). Inhibition of Photosynthetic Reactions of Isolated Intact Chloroplasts by Cadmium. J. Plant. Physiol.

[38] Zhou J., Zhang Z., Zhang Y., Wei Y., Jiang Z. (2018). Effects of lead stress on the growth, physiology, and cellular structure of privet seedlings. PLoS ONE.

[39] Sheoran I., Singh R., Abrol Y.P., Mohanty P., Govindjee (1993). Effect of heavy metals on photosynthesis in higher plants. Photosynthesis: Photoreactions to Plant Productivity.

[40] Heumann H.G. (1987). Effects of heavy metals on growth and ultrastructure of *Chara vulgaris*. Protoplasma.

[41] Mendoza-Cózatl D.G., Rodríguez-Zavala J.S., Rodríguez-Enríquez S., Mendoza-Hernandez G., Briones-Gallardo R., Moreno-Sánchez R. (2006). Phytochelatin–cadmium–sulfide high-molecular-mass complexes of *Euglena gracilis*. FEBS J..

[42] Zakrys B., Cambra-Sanchez J., Walne P.L. (2001). Chloroplast ultrastructure of *Euglena cuneata* Pringsheim, *E. deses* Ehrenberg and *E. mutabilis* (Euglenophyceae): Taxonomic significance. Acta. Protozool..

[43] Mortimer D.C., Czuba M. (1982). Structural damage to leaf chloroplasts of *Elodea densa* caused by methylmercury accumulated from water. Ecotoxicol. Environ. Saf..

[44] Narender Reddy G., Prasad M.N.V. (1990). Heavy metal-binding proteins/peptides: Occurrence, structure, synthesis and functions. A review. Environ. Exp. Bot..

[45] Morales M.E., Derbes R.S., Ade C.M., Ortego J.C., Stark J., Deininger P.L., Roy-Engel A.M. (2016). Heavy metal exposure influences double strand break DNA repair outcomes. PLoS ONE.

[46] Williams L.E., Pittman J.K., Hall J. (2000). Emerging mechanisms for heavy metal transport in plants. Biochim. Biophys. Acta-Biomembranes.

[47] Mäser P., Thomine S., Schroeder J.I., Ward J.M., Hirschi K., Sze H., Talke I.N., Amtmann A., Maathuis F.J., Sanders D. (2001). Phylogenetic relationships within cation transporter families of *Arabidopsis*. Plant. Physiol..

[48] Ortiz D., Kreppel L., Speiser D., Scheel G., McDonald G., Ow D. (1992). Heavy metal tolerance in the fission yeast requires an ATP-binding cassette-type vacuolar membrane transporter. EMBO J..

[49] Diffels J.F., Seret M.L., Goffeau A., Baret P.V. (2006). Heavy metal transporters in Hemiascomycete yeasts. Biochimie.

[50] Arunakumara K.K.I.U., Zhang X. (2008). Heavy metal bioaccumulation and toxicity with special reference to microalgae. J. Ocean. U. China.

[51] Prohaska J.R. (2008). Role of copper transporters in copper homeostasis. Am. J. Clin. Nutr..

[52] Homolya L., Varadi A., Sarkadi B. (2003). Multidrug resistance-associated proteins: Export pumps for conjugates with glutathione, glucuronate or sulfate. BioFactors.

[53] Kim D.Y., Bovet L., Maeshima M., Martinoia E., Lee Y. (2007). The ABC transporter AtPDR8 is a cadmium extrusion pump conferring heavy metal resistance. Plant J..

[54] Kolaj-Robin O., Russell D., Hayes K.A., Pembroke J.T., Soulimane T. (2015). Cation Diffusion Facilitator family: Structure and function. FEBS Lett..

[55] Ricachenevsky F., Menguer P., Sperotto R., Williams L., Fett J. (2013). Roles of plant metal tolerance proteins (MTP) in metal storage and potential use in biofortification strategies. Front. Plant. Sci..

[56] Takahashi R., Bashir K., Ishimaru Y., Nishizawa N.K., Nakanishi H. (2012). The role of heavy-metal ATPases, HMAs, in zinc and cadmium transport in rice. Plant Signal. Behav..

[57] Adle D.J., Lee J. (2008). Expressional control of a cadmium-transporting P1B-type ATPase by a metal sensing degradation signal. J. Biol. Chem..

[58] Gravot A., Lieutaud A., Verret F., Auroy P., Vavasseur A., Richaud P. (2004). AtHMA3, a plant P1B-ATPase, functions as a Cd/Pb transporter in yeast. FEBS Lett..

[59] Kim Y.Y., Choi H., Segami S., Cho H.T., Martinoia E., Maeshima M., Lee Y. (2009). AtHMA1 contributes to the detoxification of excess Zn(II) in *Arabidopsis*. Plant J..

[60] Xu Y.-H., Liu R., Yan L., Liu Z.-Q., Jiang S.-C., Shen Y.-Y., Wang X.-F., Zhang D.-P. (2011). Light-harvesting chlorophyll a/b-binding proteins are required for stomatal response to abscisic acid in *Arabidopsis*. J. Exp. Bot..

[61] Ganeteg U., Külheim C., Andersson J., Jansson S. (2004). Is each light-harvesting complex protein important for plant fitness?. Plant Physiol..

[62] Mallick N., Mohn F. (2003). Use of chlorophyll fluorescence in metal-stress research: A case study with the green microalga *Scenedesmus*. Ecotoxicol. Environ. Saf..

