# Supplementary material for: Probing the Role of the Chloroplasts in Heavy Metal Tolerance and Accumulation in Euglena gracilis
Source: Microorganisms. 2020 Jan 14;8(1):115. doi: 10.3390/microorganisms8010115 (PMC7023027; doi:10.3390/microorganisms8010115)
Supplement: Supplementary file 1 [file microorganisms-08-00115-s001.pdf]

microorganisms

Supplementary Material

Probing the role of the chloroplasts in heavy metal tolerance and accumulation in *Euglena gracilis*

Bishal Khatiwada[1,2], Mafruha T. Hasan[1,2†], Angela Sun[1,2], Karthik Shantharam Kamath[1,2,3], Mehdi Mirzaei[1,2,3], Anwar Sunna[1,2*], Helena Nevalainen[1,2*]

1 Department of Molecular Sciences, Macquarie University, Sydney NSW 2109, Australia; bishal.khatiwada@hdr.mq.edu.au (B.K); angela.sun@mq.edu.au (A.S1)
2 Biomolecular Discovery and Design Research Centre, Macquarie University, Sydney NSW 2109, Australia
3 Australian Proteome Analysis Facility, Macquarie University, Sydney NSW 2109, Australia; karthik.kamath@mq.edu.au (K.K); mehdi.mirzaei@mq.edu.au (M.M)
† Current address: School of Life and Environmental Sciences, The University of Sydney NSW 2015, Australia; mafruha.hasan@sydney.edu.au (M.H)

* Correspondence: anwar.sunna@mq.edu.au (A.S2); helena.nevalainen@mq.edu.au (H.N)

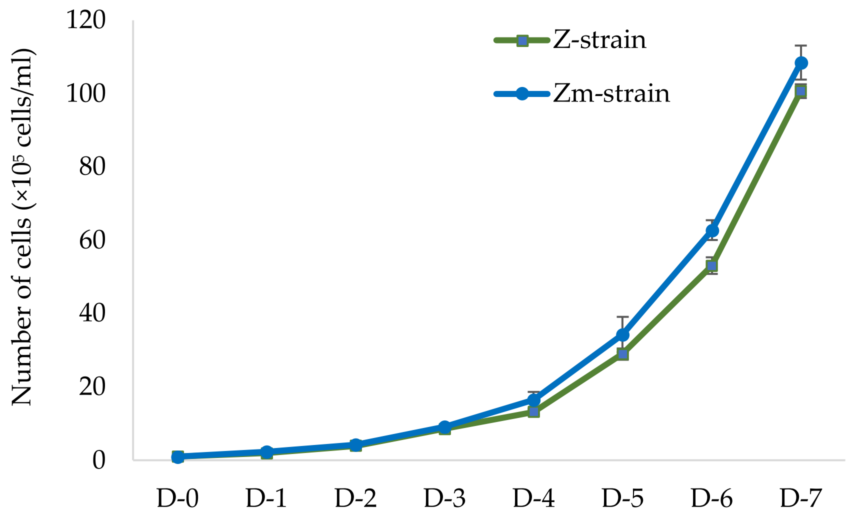

Figure S1: *E. gracilis* Z- and Zm- strains cultured in the GNY-medium only. Cells were collected each day until day 7. Error bars represent the standard deviation calculated from biological triplicates. The difference in the growth rate observed between the strains were not significantly different (p>0.05).

Table S1

Common differentially abundant proteins in the *E. gracilis* Z- and Zm- strains treated with heavy metals Cd, Hg and Pb.

**26 common proteins in Z and Zm strains treated with Cd**		
**Entry**	**UniProt ID**	**Description**
dark_m.24827	No hit	
dark_m.32100	A0A154R0T6	Outer membrane lipoprotein Blc
dark_m.7057	A0A2T4XYG7	ATP-dependent RNA helicase RhlE
light_m.23993	A0A0F0KWW7	Phenylalanine--tRNA ligase beta subunit
dark_m.44646	L8HA03	Copper translocating P-type ATPase
light_m.75809	A0A151XJ03	Dynein heavy chain 1, axonemal
light_m.60828	A0A0F0LAT1	2-dehydropantoate 2-reductase
light_m.12217	A0A0F0LPX7	Phenylacetaldehyde dehydrogenase
light_m.22749	K4CML9	No hit
light_m.54387	A0A154QKU9	Protoporphyrinogen oxidase
light_m.16210	H0ZWS8	Uncharacterized protein
dark_m.38833	A0A0M2GYA5	L-serine dehydratase TdcG
dark_m.25698	A0A154QZ68	Chaperone protein DnaJ
light_m.48058	D6WFF2	Mitochondrial inner membrane protease subunit 2-like Protein
light_m.82047	No hit	
light_m.30874	No hit	
dark_m.43765	No hit	
light_m.3606	A0A1A0FMZ0	Hybrid-cluster NAD(P)-dependent oxidoreductase
light_m.38268	No hit	

dark_m.25493	No hit	
light_m.7711	No hit	
light_m.77935	C1MMP7	Predicted protein
dark_m.57478	A0A1V9CKL2	Acetoin utilization protein
dark_m.8469	No hit	
light_m.46821	A0A202BFQ4	30S ribosomal protein S12
light_m.89541	No hit	
**4 common proteins in Z and Zm strains treated with Hg**		
light_m.55049	No hit	
dark_m.67796	A0A2T4Y663	Molybdopterin biosynthesis protein MoeB
light_m.93651	Q503L9	Nucleoredoxin
dark_m.51731	A0A2D1TGA4	Peptidase C13
**11 common proteins in Z and Zm strains treated with Hg**		
light_m.9201	L2EL58	Oxidoreductase FAD/NAD(P)-binding protein
dark_m.25956	A0A1S0X1C8	Peptidase S8
light_m.51226	No hit	
light_m.68502	G0SGH7	Protein transport protein SEC23
light_m.29375	No hit	
light_m.20070	A0A154R099	Oxidoreductase
light_m.15802	A7RFD0	Predicted protein (Fragment)
light_m.36959	A0A1E4IEZ5	Uncharacterized protein
light_m.7711	No hit	
dark_m.3544	A0A0F0LPD6	Phosphoadenylyl-sulfate reductase (thioredoxin)
light_m.65853	No hit	

Table S2

The high abundance proteins in *E. gracilis* Zm-strain during the exposure to the heavy metals Cd, Pb and Hg. Grey boxes designate the protein fold change below 1.5 during the heavy metal treatment as indicated.

			Fold Change		
**Entry**	**Uniprot ID**	**Description**	**Cd**	**Hg**	**Pb**
dark_m.60521		No hit	4.23		3.89
light_m.51226		No hit	3.31		2.99
dark_m.3993	A0A0F0L9B8	50S ribosomal protein L11	3.03		3.10
dark_m.39672		No hit	2.68		2.82
dark_m.31849	R7ZIW4	Serine hydroxymethyltransferase	2.57		3.20
dark_m.83071	A0A1S0XIL6	Thioesterase	1.93		2.38
light_m.39656		No hit	12.38	7.02	
light_m.71680	A0A202BAW4	Peptidase S41	4.79	12.58	
dark_m.47116		No hit	4.22	3.73	
light_m.51217		No hit	3.45	3.81	
dark_m.50948	A0A0S4J6N6	Presenilin	3.10	3.53	
dark_m.25493		No hit	2.93	4.89	
dark_m.48849		No hit	2.91	2.48	
dark_m.1618	A0A068RSE4	Periplasmic binding protein	2.50	3.74	
light_m.17698	P62152	Calmodulin (CaM)	2.06	1.73	
light_m.87494	L8GPK4	CPSF A subunit region protein	1.51	2.11	
light_m.39803		No hit		6.89	5.17
light_m.42333	L2ENE5	ATP-dependent zinc metalloprotease FtsH		6.85	5.08
dark_m.86441	A0A1Z1NM16	RNA recognition motif-containing protein		3.64	2.44
light_m.53310	F4Q134	Uncharacterized protein		3.27	2.33
light_m.29375		No hit		2.64	2.16
dark_m.33344	G4M004	5'-3' exoribonuclease		2.38	1.78
light_m.31954	A0A2T4XWE1	dTDP-glucose 4,6-dehydratase		1.63	1.54
dark_m.97346		No hit	5.26		
light_m.89541		No hit	4.38		
light_m.46821	A0A154QZD1	30S ribosomal protein S12	3.42		
light_m.60055	B5YMG6	Predicted protein	3.08		
light_m.84117		No hit	2.90		
light_m.86873	H3GTH9	Uncharacterized protein	2.90		
light_m.17708	D8R6L3	Uncharacterized protein	2.58		
dark_m.44646	W2ZAX1	Uncharacterized protein	2.45		
dark_m.7647		No hit	2.33		
light_m.91549		No hit	2.23		

light_m.77479		No hit	2.16	
dark_m.46264		No hit	2.12	
dark_m.69962	D0P1Q1	UDP-sugar transporter, putative	2.02	
light_m.42083	M1FT22	Multidrug resistance-associated protein member 4	1.96	
light_m.22749	K4CML9	Deleted.	1.95	
light_m.23642	Q6IFS3	Flp	1.89	
light_m.82047		No hit	1.87	
light_m.95819	U3JCF5	Bromodomain adjacent to zinc finger domain 2A	1.85	
dark_m.99125	F4Q9L9	Uncharacterized protein	1.79	
dark_m.24827		No hit	1.79	
dark_m.7057	A0A202BBA5	ATP-dependent RNA helicase rhlE	1.77	
light_m.75809	L2EF85	Putative bifunctional P-450:NADPH-P450 reductase 2	1.74	
dark_m.43765		No hit	1.72	
light_m.16210	V9G9Q8	Cu+ P-type ATPase	1.72	
light_m.12217	W7SD55	Aldehyde dehydrogenase	1.71	
light_m.17233		No hit	1.67	
light_m.35949	A8J8D7	Predicted protein (Fragment)	1.67	
light_m.22584	L2EA73	Translation initiation factor IF-2	1.67	
light_m.45620	A0A1A0FL38	CDP-diacylglycerol-serine O-phosphatidyltransferase	1.67	
light_m.54112	L2ELC0	Short chain dehydrogenase	1.64	
dark_m.51539	A0A0P0YET1	Adenylate kinase	1.61	
light_m.14317	Q39720	Cytoskeletal protein	1.60	
light_m.74679	Q4Q3D8	Uncharacterized protein	1.60	
dark_m.25698	A0A0M2HFK0	Quinone oxidoreductase 1	1.60	
dark_m.35847	A0A0F0LB15	Dehydrosqualene desaturase	1.60	
light_m.63375	Q4CRR1	Uncharacterized protein	1.59	
light_m.40585	Q63528	Replication protein A 32 kDa subunit	1.59	
dark_m.32100	A0A1A0EWG0	Outer membrane lipoprotein Blc	1.57	
dark_m.39978		No hit	1.56	
light_m.30874		No hit	1.55	
light_m.17174		No hit	1.54	
light_m.60828	A0A2D1TE80	2-dehydropantoate 2-reductase	1.54	
light_m.54387	A0A1V9CE30	Protoporphyrinogen oxidase	1.53	
light_m.61429	A0A1A0F9J5	Succinate-semialdehyde dehydrogenase	1.52	
light_m.39374	A0A1J0F973	Uncharacterized protein	1.52	
light_m.9662	Q9XIE2	ABC transporter G family member 36	1.52	
light_m.13400	H0XLU1	OVCA2, serine hydrolase domain containing		4.16

light_m.51221		No hit		3.70
dark_m.25956	A0A0F2CAY5	Extracellular serine proteinase		3.29
dark_m.62831	A0A1Z1NP99	Peptide methionine sulfoxide reductase MsrA		3.25
light_m.7720	A0A202B5N3	ABC transporter ATP-binding protein		3.09
light_m.25133	A0A1Z1NT65	Maltose O-acetyltransferase		2.74
light_m.63754		No hit		2.49
dark_m.57478	Q9BY41	Histone deacetylase 8		2.45
dark_m.50981	A0A1P8KGD3	Zinc-type alcohol dehydrogenase-like protein		2.39
light_m.69229		No hit		2.31
dark_m.3385	A0A1S0XC77	Transcriptional regulator		2.28
light_m.12682	A0A2D1TMY0	2-deoxy-D-gluconate 3-dehydrogenase		2.15
dark_m.31280	A0A0F0LPK1	3-oxoacyl-[acyl-carrier-protein] reductase FabG		2.05
light_m.9201	A0A1Z1NR96	Hydroxylamine reductase		2.00
light_m.64279		No hit		1.97
light_m.11328	A0A1Q5T7V2	Acireductone dioxygenase		1.94
light_m.21908	Q9XIE2	ABC transporter G family member 36		1.93
light_m.76103	A0A154R5B6	Molybdopterin biosynthesis protein MoeB		1.93
light_m.57952		No hit		1.90
light_m.40489	A0A1Q5SR65	Ribonuclease Z		1.89
light_m.14282	A0A0L0HMK7	Uncharacterized protein		1.80
light_m.45970	C1MSD7	Predicted protein		1.78
dark_m.48627		No hit		1.78
dark_m.79870	R7Z8D8	Zinc-type alcohol dehydrogenase-like protein		1.74
dark_m.10999	A0A1V9CNQ1	HD domain-containing protein		1.71
dark_m.34625	B5AEK8	Calcium/calmodulin dependent protein kinase II		1.68
dark_m.23482	A0A1Z1NTE8	HAD superfamily hydrolase		1.66
light_m.20245	A0A0S4IUM5	Uncharacterized protein		1.65
light_m.98107	L2EAZ8	Serine/threonine dehydratase)		1.62
light_m.55960	L1JYJ7	Uncharacterized protein		1.58
dark_m.74257	R7ZKM2	Peptidyl-tRNA hydrolase		1.58
dark_m.48875	A0A067CP42	Uncharacterized protein		1.58
dark_m.36033	K0SFY3	Uncharacterized protein		1.56
dark_m.16265	A0A202B566	Long-chain fatty acid--CoA ligase		1.55
light_m.25045	B7G487	Predicted protein		1.52
light_m.50738	A0A1A0FAG5	Pyridoxine/pyridoxamine 5'-phosphate oxidase		1.52

dark_m.12291	F1DB26	Multidrug resistance-associated protein member 2		11.82	
light_m.27912	A0A2D1TCX4	ATP-dependent RNA helicase RhlE		7.59	
light_m.83525	M1D2I9	Uncharacterized protein		6.86	
light_m.57666	A0A1A0FLF7	Translation initiation factor IF-2		4.63	
light_m.90469	A0A154QJD6	Geranyl transferase		4.50	
dark_m.31841		No hit		3.67	
light_m.55049		No hit		3.63	
light_m.66163		No hit		2.85	
light_m.20056	A0A0D0WHV6	Unplaced genomic scaffold supercont1.2		2.71	
dark_m.31843		No hit		2.54	
dark_m.78981	W7RW48	tRNA-dihydrouridine synthase		2.51	
light_m.21403	R1GGV2	Putative nucleoside diphosphatase gda1 protein		2.41	
light_m.65698	A0A1A0EW71	Cold-shock protein		2.34	
light_m.86875		No hit		2.15	
dark_m.46118	C1FFM3	Uncharacterized protein		2.08	
dark_m.21121		No hit		2.08	
light_m.10258	A0A2T4Y5S1	Methionine synthase		2.01	
light_m.81074		No hit		1.97	
dark_m.45366		No hit		1.95	
dark_m.61698	L2EKM8	Glutamate decarboxylase		1.88	
dark_m.73618	T0Q6C1	Queuosine salvage protein		1.86	
light_m.32958		No hit		1.85	
light_m.3666		No hit		1.81	
light_m.75862	B9R9H9	F-box and wd40 domain protein, putative		1.80	
dark_m.76890	A0A067BR68	Uncharacterized protein		1.72	
dark_m.50364	A0A2T4XUH8	dTDP-4-amino-4,6-dideoxygalactose transaminase		1.70	
light_m.58392	E0VMU1	Uncharacterized protein		1.68	
dark_m.27632	C1EE51	Uncharacterized protein		1.67	
light_m.8430		No hit		1.64	
dark_m.37836		No hit		1.59	
dark_m.42805	Q39758	Actin		1.59	
light_m.61516		No hit		1.58	
dark_m.58358	C5WQ78	Uncharacterized protein		1.58	
dark_m.10344	Q54DK4	Alpha-protein kinase 1		1.56	
dark_m.25768	A0A1A0F1K4	3-oxoacyl-ACP reductase		1.55	
light_m.36675	A0A1Q5TAE8	Phenylalanine--tRNA ligase alpha subunit		1.53	
light_m.31701	R0EFS4	Cytochrome P450		1.53	

light_m.93651	Q503L9	Nucleoredoxin		1.52	
dark_m.67796	A0A2T4Y663	Molybdopterin biosynthesis protein MoeB		1.51	
dark_m.50276	A0A0M2HNY1	Glucose-6-phosphate 1-dehydrogenase		1.51	
light_m.19930	A0A0F0M1E7	3-oxoacyl-[acyl-carrier-protein] synthase 2		1.51	
light_m.49430	Q9STP8	Acyl-CoA-binding domain-containing protein 2		1.50	

Supplementary Table S3

The high abundance metal binding proteins (FC >1.5) in the *E. gracilis* Z- and Zm- strains treated with heavy metals.

Entry	Protein names	Fold Change
**Z-strain**		
A0A1V2LGX5	Ribonuclease	30.26
C3ZVA2	Glutathione synthetase	14.05
A0A1Q9F763	Serine/threonine-protein phosphatase	13.73
G5CPN4	Heat shock protein Dnaj4	11.09
R0D071	Rubredoxin	9.02
Q9AR22	Magnesium-protoporphyrin IX	5.47
A0A024UDI0	Uncharacterized protein	5.37
Q21029	Mitogen-activated protein kinase kinase kinase	4.54
Q17446	Mitogen-activated protein kinase pmk-1	4.28
Q8GZR2	Cytochrome f, chloroplastic	4.28
A0A154R630	ATP-dependent zinc metalloprotease FtsH	3.81
Q8H384	Cadmium/zinc-transporting ATPase HMA3	3.65
A0A1F3CY04	Copper-translocating P-type ATPase	3.61
L2EIC4	Putative acetyl-CoA synthetase	3.57
A0A1Q9ECQ7	Ankyrin repeat domain-containing protein 50	3.55
Q9STP8	Acyl-CoA-binding domain-containing protein 2	3.51
Q9XZE5	Serine/threonine-protein phosphatase 2A catalytic subunit A	3.45
A0A067D6G0	Uncharacterized protein	3.38
A4J778	Metal reductase	3.28
W2ZAX1	Uncharacterized protein	3.25
A0A1Z1NML6	Protease III	3.10
B3G4H5	Uncharacterized protein	3.04
A0A212BY83	ATP-dependent zinc metalloprotease FtsH	3.03
A0A1Z1NV89	Beta-galactosidase	3.01
A0A2T4XZA6	Beta-galactosidase	3.00
A0A1Q5SUR7	N5-carboxyaminoimidazole ribonucleotide synthase	2.99
R7ZFQ8	tRNA-2-methylthio-N(6)-dimethylallyladenosine synthase	2.98

G5EGQ3	Serine/threonine-protein kinase max-2	2.89
L2EF85	Putative bifunctional P-450:NADPH-P450 reductase 2	2.89
A0A1E4SU43	Uncharacterized protein	2.87
A0A0Q0VXV3	Aspartate--tRNA(Asp) ligase	2.86
A0A1A0FKE0	Endonuclease V	2.83
Q5RHR0	Poly [ADP-ribose] polymerase (PARP)	2.80
A0A1Q5T4M5	Inorganic pyrophosphatase	2.79
A0A140AY28	Sodium/potassium-transporting ATPase subunit alpha	2.73
G0SGH7	Protein transport protein SEC23	2.69
A0A0D1E225	Uncharacterized protein	2.59
A0A1Q5T1Q8	Adenylate kinase (AK)	2.59
A0A0F0LTK4	Epi-isozizaene 5-monooxygenase	2.57
V9G9Q8	Cu+ P-type ATPase	2.52
A0A261XUE4	Uncharacterized protein (Fragment)	2.50
A9LNK9	30-kDa cleavage and polyadenylation specificity factor 30	2.45
A0A0W0B6R9	Amidophosphoribosyltransferase (ATase)	2.35
L1JAG4	Uncharacterized protein	2.30
W7T5H8	Phosphoglucomutase	2.30
Q8WQG9	Stress-activated protein kinase jnk-1	2.30
A0A0W0BAV8	ATP-dependent zinc metalloprotease FtsH	2.29
S2JKQ5	Uncharacterized protein	2.27
Q869N2	Serine/threonine-protein kinase pakB (dPAKb)	2.27
A0A1R0MU78	Chaperone protein DnaJ	2.25
A0A0G4IX13	Uncharacterized protein	2.23
Q74FU6	NADPH-Fe(3+) oxidoreductase subunit alpha	2.23
P11021	Endoplasmic reticulum chaperone BiP	2.22
A0A1Z1NUH4	Oligopeptidase A	2.21
A0A154R4Z0	Beta-phosphoglucomutase	2.16
A0A1Q5TAE8	Phenylalanine--tRNA ligase alpha subunit	2.14
A0A1V9YFI5	Uncharacterized protein	2.11
R0CY79	Putative Zn-dependent peptidase	2.10
K1RJM8	SAGA-associated factor 11 homolog	2.06
L2EMC1	Cytochrome P450	2.03
L2EN21	3-methyl-2-oxobutanoate hydroxymethyltransferase	1.99
R0EMH0	1-deoxy-D-xylulose-5-phosphate synthase	1.89
Q8IMX7	Mitochondrial Rho GTPase	1.89
A4H998	Putative zinc-finger protein ZPR1	1.88
Q21360	MAP kinase-activated protein kinase mak-1	1.88
R0EPZ3	ATP-dependent zinc metalloprotease	1.87
R0ERM1	NADH-quinone oxidoreductase subunit I	1.86
L2ENE5	ATP-dependent zinc metalloprotease FtsH	1.84
A0A1A0FMG5	Amidophosphoribosyltransferase (ATase)	1.83
A0A0F8BD83	Replication protein A subunit	1.82

L2ELE1	NADH-quinone oxidoreductase subunit B	1.82
A1AL80	Rubrerythrin	1.76
A0A0W0B7C7	Chaperone protein DnaJ	1.66
A0A061G729	Calcium-binding EF-hand family protein	1.66
A0A0Q0VNU4	Flap endonuclease 1	1.62
A0A0F2CCA7	Chaperone protein DnaJ	1.62
A0A0M2HRQ1	ATP-dependent zinc metalloprotease FtsH	1.60
A0A0F2C6T0	Aminodeoxyfutalosine deaminase	1.60
E0CT66	Uncharacterized protein	1.58
L2EM15	Phosphoenolpyruvate carboxykinase	1.57
G7HXR1	AraC-family transcriptional regulator	1.51
A0A212BMG8	3-methylcrotonyl-CoA carboxylase	1.50
A0A2P6TKR5	Host cell factor 1	3.01
A0A0S4JP31	Adenylosuccinate synthetase	1.76
A0A0F2CAY5	Extracellular serine proteinase	2.57
A0A1Z1NNS6	Phenylalanine-4-hydroxylase	2.26
A0A1Z1NR96	Hydroxylamine reductase	2.90
A0A0W0BA55	Carbonic anhydrase	2.05
**Zm-strain**		
A0A2R5GHD4	Copper-transporting ATPase RAN1	7.59
L2ENE5	ATP-dependent zinc metalloprotease	5.08
A0A0F2CAY5	Extracellular serine proteinase	3.29
H3GTH9	Uncharacterized protein	2.90
W2ZAX1	Uncharacterized protein	2.45
Q9BY41	Histone deacetylase 8	2.45
A0A1P8KGD3	Zinc-type alcohol dehydrogenase-like protein	2.39
C1FFM3	Uncharacterized protein	2.08
P62152	Calmodulin (CaM)	2.06
A0A2T4Y5S1	Methionine synthase	2.01
A0A1Z1NR96	Hydroxylamine reductase	2.00
A0A1Q5T7V2	Acireductone dioxygenase	1.94
A0A1Q5SR65	Ribonuclease Z (RNase Z)	1.89
U3JCF5	Bromodomain adjacent to zinc finger domain 2A	1.85
B9R9H9	F-box and wd40 domain protein, putative	1.80
R7Z8D8	Zinc-type alcohol dehydrogenase-like protein	1.74
L2EF85	Putative bifunctional P-450:NADPH-P450 reductase 2	1.74
V9G9Q8	Cu+ P-type ATPase	1.72
Q54DK4	Alpha-protein kinase 1	1.56
A0A1Q5TAE8	Phenylalanine--tRNA ligase alpha subunit	1.53
R0EFS4	Cytochrome P450	1.53
Q9STP8	Acyl-CoA-binding domain-containing protein 2	1.50
